# Financial Health After Private Equity Hospitals Are Sold

**DOI:** 10.1001/jamahealthforum.2025.3217

**Published:** 2025-08-08

**Authors:** Sneha Kannan, Zirui Song

**Affiliations:** 1 Department of Critical Care Medicine, University of Pittsburgh, Pittsburgh, Pennsylvania; 2 Department of Health Care Policy, Harvard Medical School, Boston, Massachusetts; 3 Department of Medicine, Massachusetts General Hospital, Boston; 4 Center for Primary Care, Harvard Medical School, Boston, Massachusetts; 5 Associate Editor, *JAMA Health Forum*

## Abstract

This cohort study evaluates early evidence of hospital outcomes after secondary private equity acquisitions.

## Introduction

Policymakers are investigating private equity (PE) acquisitions of hospitals.^1^ Recently, however, many hospitals have been sold (divested) by their initial PE owners to another PE firm or corporate owner. Hospital outcomes after such sales remain unknown. We examined changes in hospital financial health after sale, contextualized with analysis of initial PE acquisitions.

## Methods

Using 2006-2022 Medicare Cost Report data, we compared 18 PE hospitals sold to second PE firms (exposure) with 18 PE hospitals sold to non-PE, for-profit firms (comparison). All hospitals were initially acquired by PE firms between 2005 and 2018 and sold between 2009 and 2020. We then compared 242 initial PE acquisitions relative to 870 nonacquired control hospitals.^2,3^

For the 18 secondary and 242 initial PE acquisitions, we assessed mean changes in operating margins, total revenues per available patient bed-day, and total expenses per available patient bed-day relative to the control group from 3 years before to 3 years after acquisition using a difference-in-differences design and an ordinary least-squares regression model. Acquisitions, matching, and statistical analyses are further detailed in the eMethods in Supplement 1. This study was approved by the Harvard Institutional Review Board. Analyses were conducted using Stata version 18 (StataCorp).

## Results

After PE hospitals were acquired by a second PE owner, operating margins decreased by 8.4 percentage points (95% CI, −15.8 to −1.0) relative to PE hospitals sold to non-PE, for-profit owners (Figure, A). This narrowing of the operating margin was explained by a mean increase of $316 (95% CI, 15 to 618) in hospital expenses per available bed-day—ie, increased spending as opposed to cost cutting—relative to PE hospitals sold to non-PE, for-profit owners (Figure, B). There was no differential change in revenue per available inpatient day between the 2 groups.

**Figure.**
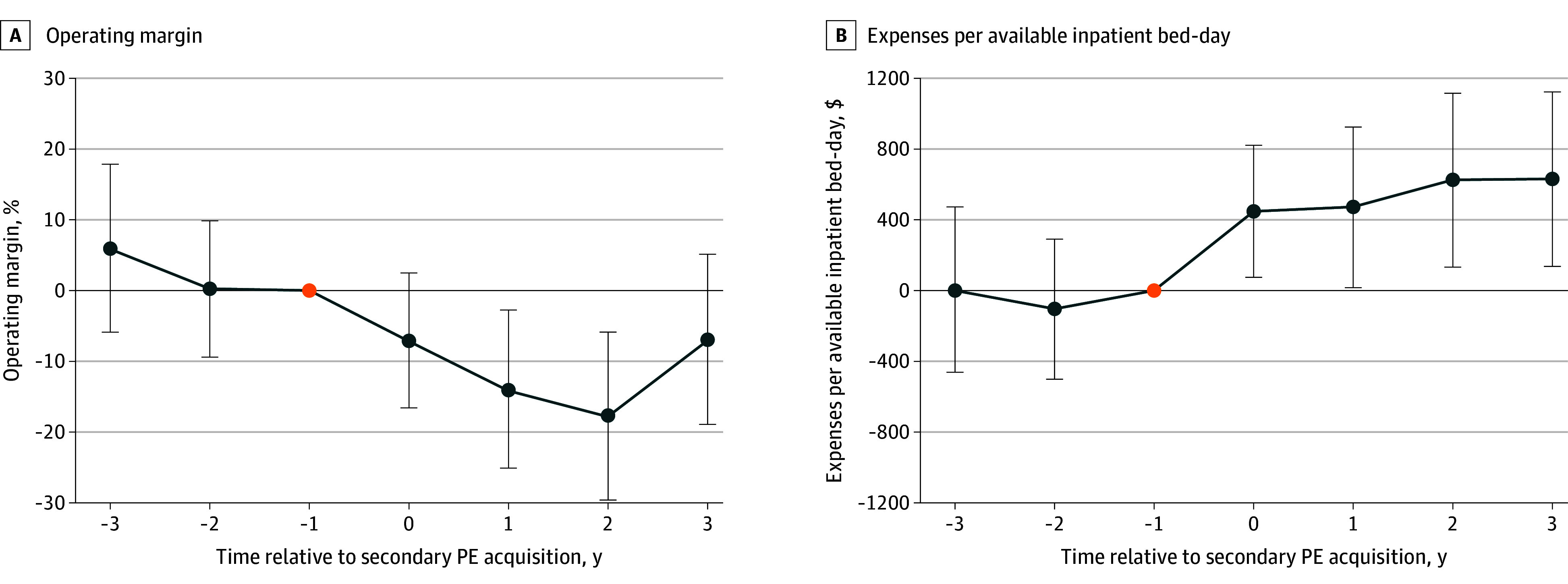
Changes in Operating Margin and Expenses Associated With Sale of Private Equity (PE) Hospitals to a Second PE Firm, Relative to Other For-Profit Firms Event study plots depict the differential changes in operating margin (A) and expenses (B) among PE hospitals sold to a second PE firm relative to those sold to a for-profit, nonprivate equity firm. Error bars indicate 95% CIs.

In contrast, the initial PE acquisition of hospitals was not associated with increased hospital expenses. Rather, hospital expenses suggested a mean decline of $305 (95% CI, −874 to 263) per available bed-day, but this was not statistically significant. A mean reduction in revenues of $248 (95% CI, −780 to 283) per available bed-day was also not statistically significant.

However, after excluding HCA Healthcare hospitals (where the initial PE owners remain the plurality shareholders), initial PE acquisition was associated with a statistically significant mean reduction in expenses of $586 (95% CI, −998 to −176) per inpatient bed-day—consistent with cost cutting—with a similar, though nonsignificant, reduction of $624 (95% CI, −1355 to 107) in revenues per inpatient bed-day.^3,4^

## Discussion

In this sample of 36 hospitals sold by their initial PE owners, those sold to a second PE firm increased hospital expenditures, which reduced operating margins, relative to peers sold to non-PE firms. In contrast, the initial PE acquisition (outside of HCA Healthcare hospitals) was followed by cuts in hospital expenditures, which likely reflected staffing reductions.^4^

This indicates that, on average, a second PE owner managed hospitals differently than other for-profit firms that buy hospitals from the initial PE owner.^2^ Notably, secondary PE owners did not further cut costs after prior cost cutting by the first owner.^2^ Instead, they were more likely to reverse some of the prior cost cutting by raising spending, which may span labor and supplies, capital expenditures (like real estate leases), or administrative costs.

Increased spending on staffing may improve patient outcomes, whereas spending on leases or administrative expenses may not. To maintain service lines and staffing levels to help preserve access to care, policymakers could consider requiring funds to be set aside from PE owners of hospitals (a type of escrow account), which would help ensure that hospitals can pay vendors and continue operations uninterrupted through subsequent acquisitions.^5,6^ As PE-owned hospitals are increasingly sold, the impact on patients and communities deserve monitoring. Limitations include a small sample size of acquisitions. These acquisitions are not representative of all such secondary sales by private equity and may have led to outliers having an outsize effect on our estimates.

## Notes

Supplement 1.
eMethods.

Supplement 2. Data Sharing Statement
