# Supplementary material for: Financial Health After Private Equity Hospitals Are Sold
Source: JAMA Health Forum. 2025 Aug 8;6(8):e253217. doi: 10.1001/jamahealthforum.2025.3217 (PMC12334948; doi:10.1001/jamahealthforum.2025.3217)
Supplement: Supplement 1. — eMethods. [file jamahealthforum-e253217-s001.pdf]

Supplemental Online Content

Kannan S, Song Z. Financial health after private equity hospitals are sold. *JAMA Health Forum.* 2025;6(8):e253217. doi:10.1001/jamahealthforum.2025.3217

eMethods.

This supplementary material has been provided by the authors to give readers additional information about their work.

© 2025 Kannan S et al. *JAMA Health Forum*.

eMethods.

Private equity-acquired hospitals and subsequent sales and buyers were identified using a variety of sources, consistent with the literature. These included S&P Global Market Intelligence Software, Merger and Acquisition reports by Irwing Levin Associates, U.S. Securities and Exchange Commission filings, Pitchbook, and press releases.[1,2]

Among 287 acute care hospitals newly acquired by private equity before 2019,[1] 213 (74%) are still majority owned by their initial private equity firms today. This is driven by the two largest acquisitions (by number of acquired hospitals): Hospital Corporation of America Healthcare (HCA), in which the initial private equity investor group continues to be the largest shareholder after HCA's 2011 initial public offering (IPO),[3,4] and the Lifepoint acquisition in 2018, which remains held by Apollo Global Management. The remaining 74 hospitals were sold to a second private equity firm (32%), to a non-private equity for-profit owner (46%), or to a non-profit owner (16%), with 6% having closed under private equity ownership.

We compared private equity hospitals sold to a second private equity owners to private equity hospitals sold to non-private equity, for-profit firms. To do this, we excluded hospitals still owned by the initial private equity firm, those with missing data, and closures, as well as the very few hospitals sold to non-profit firms. These criteria resulted in 36 hospitals. All began as non-private equity hospitals, were acquired by private equity for the first time between 2005-2018, and sold between 2009-2020. Eighteen (50%) were sold to another private equity firm, while the other 18 hospitals (50%) were sold to for-profit, non-private equity owners. Characteristics of these hospitals are shown in eTable 1.

Using 2006-2022 Medicare Cost Report data, we evaluated hospital operating margins, total revenues, and total expenses for these secondary acquisitions using a stacked approach consistent with prior work.[2,5] Hospital operating margins are a measure of profitability and are calculated by dividing operating profit (operating revenues minus operating costs) by operating revenue. Operating margins vary widely for hospitals; they can be negative if operating revenue or cash outflows (expenses) exceed revenue inflows.[6] Revenues included payments generated from patient care, and expenses included costs

© 2025 Kannan S et al. *JAMA Health Forum*.

of delivering care. Both revenues and expenses were indexed to available patient bed days to account for hospital bed size and throughput.

Hospital financial outcomes were aligned by year relative to sale of hospitals to a second private equity firm (defined as the "exposure" of interest) and to non-private equity, for-profit firms. Hospitals contributed up to 3 years of data before and after the exit. We used an ordinary least squares regression model, in which outcomes for a hospital in a given year were regressed on indicators for exposure, event year, and the interactions between exposure and event year. The estimated effect of sale to a second private equity firm relative to a non-private equity buyer was the mean of the changes in outcomes across post-acquisition years relative to the pre-acquisition baseline in the exposed group relative to the control group. We adjusted for the number of beds in a hospital, along with fixed effects for hospital and year.

Several newer difference-in-differences and event study estimators were less appropriate in this context, as do not allow for control units to undergo staggered adoption of an intervention or exposure.[7-10] Instead, these newer estimators compare treated units to all control units (specified by the researcher to be either never-treated or not-yet-treated) in all years relevant to the treated units.

In secondary analyses, we reexamined the larger cohort of *initial* hospital acquisitions by private equity firms (hospitals acquired by private equity from a non-private equity owner for the first time), to provide a benchmark for interpreting the estimated effects of the sale to a second private equity owner. As in prior work, each hospital initially acquired by private equity firms was matched with up to 8 non-private equity (control) hospitals using exact matching on year, ownership type, teaching status, and Census region, with cardinality matching on total beds.[8] This cohort included 242 initial private equity-acquired hospitals and 870 matched controls. We conducted this analysis of initial hospital acquisitions with and without the large Hospital Corporation of America (HCA) acquisition, which was shown to increase prices rather than cutting costs. Characteristics of these initial acquisitions are shown in eTable 1.

© 2025 Kannan S et al. *JAMA Health Forum*.

References

1. Kannan S, Song Z. Financial and Clinical Characteristics of Hospitals Targeted by Private Equity Firms. JAMA Intern Med. 2024 Sep 1;184(9):1127-1129.
2. Bruch JD, Gondi S, Song Z. Changes in Hospital Income, Use, and Quality Associated With Private Equity Acquisition. JAMA Intern Med. 2020;180(11):1428-1435.
3. HCA Holdings Inc. Form 424B4-Prospectus. Securites and Exchange Commission. Filling Date March 11, 2011. Last accessed November 14, 2024. https://www.sec.gov/Archives/edgar/data/860730/000095012311024716/0000950123-11-024716-index.htm
4. Form Def 14A. Filer HCA Healthcare. Securities and Exchange Commission. Filing Date March 10, 2023. Last accessed November 14, 2024. http://pdf.secdatabase.com/35/0001193125-23-067645.pdf
5. Kannan S, Bruch JD, Song Z. Changes in Hospital Adverse Events and Patient Outcomes Associated With Private Equity Acquisition. JAMA. 2023;330(24):2365-2375.
6. National Hospital Flash Report. Kaufman Hall. Published February 2023. Last accessed November 14, 2024. https://www.kaufmanhall.com/sites/default/files/2023-02/KH-NHFR_2023-02.pdf
7. Rios-Avila F, Sant'Anna P, Callaway B. CSDID: Stata module for the estimation of Difference-in-Difference models with multiple time periods. Statistical Software Components S458976, Boston College Department of Economics. Published in Boston, MA; Updated 25 Feb 2023. Accessed November 13, 2024. https://econpapers.repec.org/software/bocbocode/s458976.htm
8. Sun L, Abraham S. Estimating dynamic treatment effects in event studies with heterogeneous treatment effects. Journal of Econometrics. 2021;225:175-199.
9. Wooldridge, Jeffrey M., Two-Way Fixed Effects, the Two-Way Mundlak Regression, and Difference-in-Differences Estimators (August 17, 2021). http://dx.doi.org/10.2139/ssrn.3906345
10. Cengiz D, Dube A, Lindner A, Zipperer B, The Effect of Minimum Wages on Low-Wage Jobs, The Quarterly Journal of Economics. 2019;134(3):1405-1454.

© 2025 Kannan S et al. *JAMA Health Forum*.
