# Supplementary material for: Financial Health After Private Equity Hospitals Are Sold
Source: JAMA Health Forum. 2025 Aug 8;6(8):e253217. doi: 10.1001/jamahealthforum.2025.3217 (PMC12334948; doi:10.1001/jamahealthforum.2025.3217)
Supplement: Supplement 2. — Data Sharing Statement [file jamahealthforum-e253217-s002.pdf]

Data Sharing Statement

Kannan. Financial Health After Private Equity Hospitals Are Sold. *JAMA Health Forum*. Published August 08, 2025. doi:10.1001/jamahealthforum.2025.3217

Data
Data available: No

Additional Information
Explanation for why data not available: Per the data use agreement with Harvard Medical School.
